# Pyorrhea and Its Reflexes

**Published:** 1914-10-15

**Authors:** R. L. Tower


### PYORRHEA AND ITS REFLEXES.

R. L. TOWER, D.D.S.

The tremendous gap which has been existing between Dentistry and Medicine for years is beginning to be eliminated by the wide-awake members of each profession. A

great many men have contended themselves with cavity preparation, local treatment, as necessary, and the finale being a filling of whatsoever it seemed best to use; many times silver, amalgam or porcelain, according to the cosmetic effect desired or the whims of the patient.

With the rapid strides made in medicine the past ten years, notably with the internal secretion and their reflexes, the relationship of tooth abnormalities, viz.: Pyorrhea, dental caries, abscess roots, etc., becomes more evident to that of the general health. In fact, I believe if people were in perfect health there would be a very small percentage of the above-named ailments, as compared to the present conditions. That we, as dentists, must keep uppermost in our minds that there is a relationship between tooth decay, Pyorrhea, and the various changes in the body governing the general health. The time is rapidly approaching that whenever a case presents itself with the diseased condition of the teeth or the gums, commonly called Pyorrhea, a searching examination will be made concerning the general health, and, if possible, the reason thereof definitely ascertained. We must begin by removing the cause and then build up. I believe that most cases of Pyorrhea, if not all, are but the manifestation of some abnormal condition of the body. Dr. Black has proven that we will not have Calculus, or Tartar, gather on our teeth unless we eat more food than can be used by the system. Most people eat too much, and nature throws off the surplus, some of which is in the saliva, and is then deposited on the teeth with the food that is not removed. The food products and the saliva mix and begin to harden in twenty-four to forty-eight hours if not removed. The condition of the system is also weakened by taking too much food as well, and the bodily resistance lowered, and together with the irritating effect of the deposits causes an inflammation, which opens a field for bacteria to gain a foothold, Pyorrhea resulting. Therefore, I say Pyorrhea is the result of an unbalanced system. It may be from improper food as well as too much, and is usually years in reaching the stage called Pyorrhea.

Auto-intoxication, or auto-infection, due to the lack of proper elimination of the poisons and waste products of the body, is responsible for innumerable diseases of the body, and before we can begin to accomplish much in the treatment of Pyorrhea we must tone up the excretory organs and regulate the diet. "If the ship springs a leak the pumps may be able to take care of the water until she reaches port, but unless the leak is stopped it would not be safe to start on another voyage." So it is with our bodies. Unless we remove the cause of trouble our efforts will only be palliative and the condition will return and our efforts will be of no avail.

In support of the above contention and that various conditions affecting the fifth nerve are largely due to reflections, the following case is cited:

History—The patient in question is of middle age; one who takes more than the usual care of her health. Suffering from acne rosacea of both alea of the nares, consulted a specialist. Observing an existing case of Pyorrhea with a number of decayed teeth, he fully realized that this was a reflex condition, and after a thorough examination was requested to call on me for treatment. The nerve relationship between the teeth and nose was explained to the patient and a complete cleaning of the oral cavity advised.

The patient presented a chronic case of Pyorrhea, which involved every tooth of upper and lower jaws; the pus seepage being tremendous from the first molars of both inferior and superior Maxillae. One should note that the inferior teeth had been treatd six months previous, and unquestionably this Pyorrhea existed at this time and was not treated.

The roots of nearly all the teeth were covered with salivary and serumal calculus, extending well up under the gums, causing very deep Pyorrhea pockest by its irritating effect. In this case the treatment consisted of a thorough scaling of the roots of a surgical nature, requiring several sittings, followed three times a week with a local astringent application to the pockets and gums. The action of the local medical treatment was enhanced by the action of the High Frequency Electric Current for a period of thirty minutes. The current has a very stimulating effect on cell growth and the circulation of the blood locally.

The following two weeks' treatment, as outlined above, resulted in an entire disappearance of all pus. The first molars of the lower jaw did not tighten, the roots having been considerably absorbed at their apices. The lower right molar was removed in trying to cut the crown off. The ill-fitting crown and cavity underneath, extended disto-bucco-mesially. At the end of six weeks' treatment, and following the removal of the molar tooth, there has been an entire disappearance of the acne rosacea of the nares. The gums at this time still showed some congestion, but were much improved. Prescribed laxative and antacid treatment, since which time patient's gums have improved steadily and are now in apparently healthy condition.

Often the matter of finance is too frequently taken into consideration by the dentist, for fear that the patient may go elsewhere. Nevertheless, the patient should be fully informed of his condition, and in so doing we have fulfilled our duty to the patient.

The patient had a feeling of fullness in the right ear, diagnosed as sub acute inflammation of the eustachian tube. This condition has nearly all passed away since the treatment of the Pyorrhea began. It is remarkable that treating the Pyorrhea cleared up a so mewhat remote trouble, undoubtedly reflex.

The relation between these conditions and the Pyorrhea can be attributed to auto-intoxication, brought about by swallowing of the pus and decayed food products, thereby causing a general low grade infection and lowering of vitality in the terminal tissues, and by nerve irritation through the branches of the fifth nerve. When the bodily resistance is lowered, the pus-producing germs find an easier time gaining an entrance and rapidly increasing continue their havoc by the rapid breaking down of the surrounding tissue. We cannot be healthy when pus is flowing from tissues and being absorbed into our system. Yet hundreds of mouths are in just this condition, only requiring time for the completion of the pathological function (Pyorrhea) involved in this case.

There are many derangements of the general system which require the aid of the dentist, as well as conditions of the mouth, where the physician can be of service to the dentist, as, for instance, a case of Pyorrhea, aggravated, if not entirely caused, by high blood pressure.

According to Talbot, high blood pressure, with toxic products, circulating in the blood, will set up an inflammation in the alveolar process due to the expansion of the arteries.

Many cases of this character are to be found, and I believe we are coming to require a better knowledge of the general system in treating diseases of the oral cavity. By more frequent consultation between the Dentists and Physicians regarding cases in practice, we will be the better able to render efficient service to humanity.—*Northwest Journal of Dentistry.*

